# Coupled motions of the spine under standardized *in vitro* conditions: a systematic review and meta-analysis

**DOI:** 10.3389/fbioe.2025.1686524

**Published:** 2025-11-12

**Authors:** Christian Liebsch, Hans-Joachim Wilke

**Affiliations:** Institute of Orthopaedic Research and Biomechanics, Trauma Research Centre Ulm, Ulm University Medical Centre, Ulm, Germany

**Keywords:** spine, coupled motions, *in vitro*, biomechanics, systematic review, meta-analysis

## Abstract

**Introduction:**

Coupled motions are defined as motions outside the primary motion plane and are used for *in vivo* kinematic measurements as well as validation of experimental and numerical models of the spine. Owing to differences in the individual movement comforts of participants and imprecise measurement methods, previous *in vivo* studies have been unable to determine distinct patterns of coupled motions. The aim of this meta-analysis was to identify reproducible coupled motion patterns from *in vitro* studies with standardized loading conditions for each section of the spine.

**Methods:**

A systematic literature search was conducted in the PubMed, Web of Science, and Embase databases in accordance with the PRISMA guidelines to identify *in vitro* studies on the coupled motions of human specimens (n = 120). In a three-stage procedure, we excluded all studies except those that allowed quantitative comparability of coupled motions in the individual loading directions (n = 20). The inclusion criteria were testing the intact state, quasistatic flexibility measurements using pure moments, and specifications of the analyzed levels. The coupled motions were calculated as values relative to the primary range of motions and quantitatively evaluated via meta-analysis. The one-sample Wilcoxon signed-rank test was performed in SPSS to determine the reproducibility of the coupled motions for each segmental level.

**Results:**

Overall, no relevant coupled motions were identified for primary flexion and extension (*p* > 0.05). For primary lateral bending, there was evidence for low extension and moderate-to-high ipsilateral axial rotation in the thoracic spine as well as moderate ipsilateral axial rotation in the subaxial cervical spine (*p* < 0.05). For primary axial rotation, there were reports on low-to-moderate contralateral lateral bending in the thoracic spine and high-to-dominant ipsilateral lateral bending in the subaxial cervical spine (*p* < 0.05).

**Discussion:**

This meta-analysis of *in vitro* studies identified some characteristic coupled motion patterns of the spine, specifically a strong motion coupling interrelationship between lateral bending and axial rotation. More studies are required to extend and substantiate the findings of this meta-analysis. Nevertheless, this dataset is valuable for validating experimental and numerical studies of the spine as well as interpreting the coupled motion behaviors of the passive spinal structures.

## 1 Introduction

Coupled motions are also referred to as secondary or out-of-plane motions and are defined as motions about axes that are associated with a simultaneous primary motion about another axis. With regard to the spine, the causes and effects of coupled motions have been investigated extensively and discussed widely in the past. Early *in vivo* and *in vitro* investigations by the orthopedic surgeon Robert W. Lovett in 1900 already suggested strong motion coupling behaviors between lateral bending and axial rotation (Lovett, 1900). While Lovett hypothesized that spinal coupled motions play a role in the etiology of idiopathic scoliosis (Lovett, 1905), further *in vivo* studies reported abnormally increased coupled motions in patients with degenerative lumbar scoliosis (Xu et al., 2024) as well as in patients suffering from lower-back pain (Weitz, 1981; Pearcy et al., 1985; Parnianpour et al., 1988; Stokes et al., 1981; Ochia et al., 2006). Coupled motions have been used to characterize the *in vivo* three-dimensional motion behaviors in patients receiving surgical treatment on the cervical spine (Lee et al., 2014). However, *in vivo* studies have limitations for the evaluation of coupled motions owing to the individual movement comforts of the participants and potential imprecisions in the measurement techniques used, which have consequently resulted in conflicting findings. Thus, previous literature reviews of *in vivo* studies were unable to determine consistent coupled motion characteristics of specific spinal sections (Harrison et al., 1998; Legaspi and Edmond, 2007; Sizer et al., 2007). Another drawback of *in vivo* studies is that the roles of the passive spinal structures in coupled motions cannot be determined clearly as spinal loading is generated by complex muscle forces. *In vitro* studies using standardized loading conditions can provide more reproducible coupled motion data, which are required for distinct interpretations of the *in vivo* findings as well as validation of the experimental and numerical spine models regarding three-dimensional kinematics. Since the extant *in vitro* studies have predominantly investigated single spinal sections or segment levels, a comprehensive overview of the coupled motion characteristics of the entire spine is yet to be developed. Hence, the aim of the present systematic literature review and meta-analysis is to collate and compare coupled motion data of the spine from *in vitro* studies performed under standardized loading conditions.

## 2 Methods

### 2.1 Systematic literature review

**FIGURE 1:**
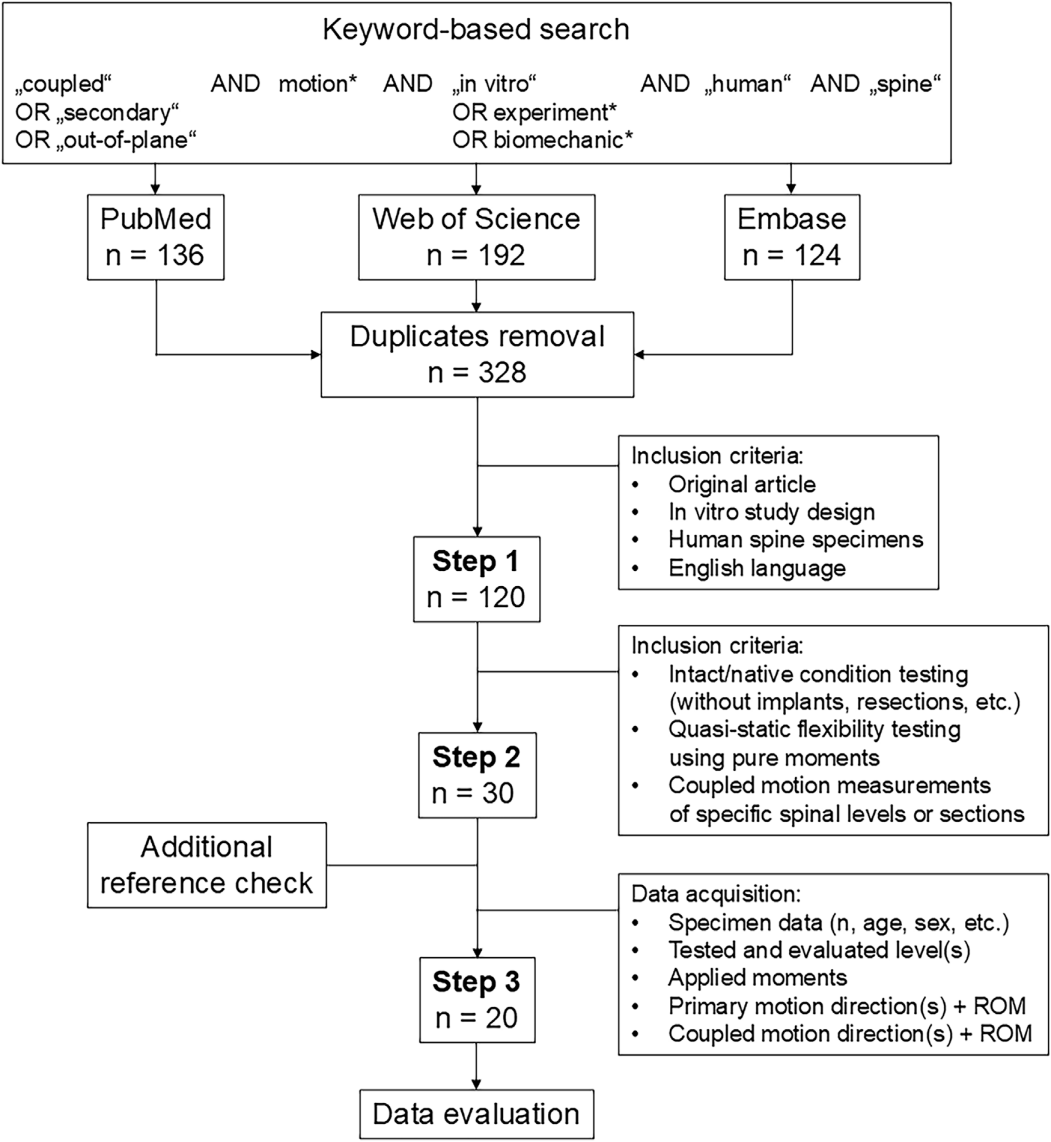
Flow diagram illustrating the systematic literature search according to the PRISMA guidelines (Page et al., 2021).

Based on the Preferred Reporting Items for Systematic reviews and Meta-Analyses (PRISMA) guidelines (Page et al., 2021), we conducted a keyword-based literature search on the PubMed, Web of Science, and Embase medical research databases in April 2025 to identify *in vitro* studies reported on the coupled motions of the human spine (Figure 1). After removing duplicates using EndNote 21.5 (Clarivate Analytics, Philadelphia, PA, United States), n = 328 studies were collected. In the first selection step, we included all articles that reported (1) original data; (2) an *in vitro* study design; (3) the use of human spine specimens and (4) were written in English language, which resulted in n = 120 articles for closer examination. In the second selection step, the inclusion criteria were defined such that (5) biomechanical data on the intact specimen condition had to be reported (i.e., a testing condition without any implants, resections other than those related to specimen preparation, etc.); (6) quasistatic flexibility testing had to be performed via pure moments (i.e., no eccentric loading, loading without any additional compressive or follower loading, no constraints or shear forces, etc.) according to well-accepted recommendations for spinal *in vitro* testing (Wilke et al., 1998), and (7) the segmental levels or spinal sections on which the coupled motion measurements were performed had to be specified. On the resulting n = 30 articles from this step, we checked the reference sections for potential publications to be included and data on the specimens (sample size, donor age and sex, etc.), in addition to acquiring information on the tested and evaluated segmental level(s), applied moments, as well as motion directions and range of motion (ROM) of the primary and coupled motions. As some of the articles reported only the ranges of coupled motions but not the primary motions (Oxland et al., 1992), ROM data of intact specimens that were already published in prior studies (Cholewicki et al., 1996; Liebsch et al., 2020a), ratios but not directions of the coupled motions (Puttlitz et al., 2004; Yoganandan et al., 2008; Mannen et al., 2015a; Mannen et al., 2015b), ROM data of non-specified segmental levels (Winkelstein and Myers, 2002; Brasiliense et al., 2011), or combined primary and coupled motions (Daniels et al., 2012), such works were excluded to obtain n = 20 studies for the final data evaluations.

### 2.2 Data evaluation

The ROM data of the coupled motions were acquired either directly as percentage values of the respective primary ROMs from the published text (if reported) or via calculations from the absolute mean or median values of the primary and coupled ROMs. If the data were illustrated as diagrams, the single data points were digitized using an open-source software for image analysis (Engauge Digitizer 12.1). All obtained data were then collated and postprocessed using Excel version 2504 (Microsoft Corp., Redmond, WA, United States). For our meta-analysis, the coupled motion data were divided into individual secondary motion planes for each primary motion direction, which resulted in six sub-meta-analyses, namely, coupled lateral bending and coupled axial rotation during primary flexion/extension, coupled flexion/extension and coupled axial rotation during primary lateral bending, as well as coupled flexion/extension and coupled lateral bending during primary axial rotation. To classify the magnitudes of the coupled motions relative to their respective primary motions, percentage values below 20% were defined by the authors as low coupled motions, values between 20% and 50% were defined as moderate coupled motions, values between 50% and 100% were designated as high coupled motions, and values over 100% were assigned as dominant coupled motions. Additionally, to determine the reproducibility of the findings, the non-parametric one-sample Wilcoxon signed-rank test was performed on the percentage data of each segmental level using SPSS version 29 (IBM Corp., Armonk, NY, United States) to determine the statistical difference of the relative coupled ROM values from zero for each segmental level and primary motion direction; p-values greater than or equal to 0.1 were defined as non-reproducible, while values between 0.5 and less than 0.1 were deemed to have a tendency toward reproducibility, and those below 0.05 were considered reproducible, following the recommendations of Bland (1986).

## 3 Results

### 3.1 Overall collective

Of the 20 *in vitro* studies included in this meta-analysis (Table 1), eleven reported the coupled motions of cervical, seven reported the coupled motions of thoracic, and four reported the coupled motions of lumbar spinal sections or segmental levels. The first *in vitro* study on coupled motions was published by Panjabi et al. (1989) from Yale University on the lumbar spine. This and further investigations were carried out at a total of six institutions: Yale University, New Haven, CT, United States (Panjabi et al., 1993a; Panjabi et al., 1994; Panjabi et al., 2001); ENSAM/Arts et Métiers ParisTech, Paris, France (Wen et al., 1993; Barrey et al., 2009; Barrey et al., 2015; Taverne et al., 2024); Edward Hines Jr VA Hospital, Hines, IL, United States (Patwardhan et al., 2012; Muriuki et al., 2024); St. Joseph’s Hospital and Medical Center, Phoenix, AZ, United States (Colle et al., 2013); Ulm University, Ulm, Germany (Liebsch et al., 2017; Liebsch et al., 2018; Liebsch et al., 2020b; Liebsch et al., 2020c; Wilke et al., 2020; Liebsch et al., 2024; Vogt et al., 2024a; Vogt et al., 2024b); Drexel University, Philadelphia, PA, United States (Orbach et al., 2023). Every study included in this meta-analysis reported the testing of at least n = 6 specimens. The mean donor age of the tested specimens (reported in 17 studies) ranged between 28 and 81 years; the donor sex (reported in 16 studies) was roughly equally distributed in nine studies, while more than two-thirds of the specimens originated from male donors in four studies and more than two-thirds of the specimens originated from female donors in three studies. The applied loads ranged from 1 Nm to 4.5 Nm for the cervical, 2.5 Nm to 8 Nm for the thoracic, and 5 Nm to 10 Nm for the lumbar spinal specimens.

**TABLE 1:** Overview of the studies included in this meta-analysis (n.a. = not available).

Study	Year	Age ± standard deviation (range) in years	Sex	Tested levels	Evaluated levels	Applied load
Panjabi et al. (1989)	1989	n.a.	n.a.	L1–S1 (n = 6)	L1–L2, … , L5–S1	10 Nm
Panjabi et al. (1993a)	1993	44 (35–53)	3 f, 4 m	C0–C3 (n = 7)	C0–C1, C1–C2	1.5 Nm
Wen et al. (1993)	1993	66 ± 10 (44–87)	n.a.	C2–C3 (n = 11), C3–C4 (n = 9), C4–C5 (n = 15), C5–C6 (n = 8), C6–C7 (n = 13)	C2–C3, … , C6–C7	1.4–4.5 Nm
Panjabi et al. (1994)	1994	51 (35–62)	9 m	L1–S1 (n = 5), L2–S1 (n = 4)	L1–L2, … , L5–S1	10 Nm
Panjabi et al. (2001)	2001	n.a.	n.a.	C0–C5 (n = 1), C0–C6 (n = 5), C0–C7 (n = 2), C2–C7 (n = 8)	C0–C1, … , C6–C7	1 Nm
Barrey et al. (2009)	2009	69 ± 5 (54–74)	3 f, 3 m	C4–C6 (n = 6)	C4–C6	1.6 Nm
Patwardhan et al. (2012)	2012	51 ± 5 (42–56)	6 f, 6 m	C3–C7 (n = 12)	C5–C6	1.5 Nm
Colle et al. (2013)	2013	56 (45–64)	1 f, 8 m	C3–T1 (n = 9)	C5–C6	1.5 Nm
Barrey et al. (2015)	2015	62 ± 6 (55–77)	6 f, 6 m	C2–T2 (n = 12)	C3–C7	2 Nm
Liebsch et al. (2017)	2017	56 (50–65)	5 f, 1 m	C7–L1 (+ rib cage, n = 6)	T1–T12	2 Nm
Liebsch et al. (2018)	2018	54 ± 6 (40–60)	1 f, 7 m	C7–L1 (+ rib cage, n = 8)	T1–T2, … , T11–T12, T1–T12	5 Nm
Liebsch et al. (2020b)	2020	81 (63–99)	2 f, 4 m	C7–L1 (+ rib cage, n = 6)	T3–T4, T4–T5, T7–T8, T8–T9, T1–T12	5 Nm
Liebsch et al. (2020c)	2020	56 ± 7 (40–68)	8 m	T1–T2 (n = 8), T3–T4 (n = 8), T5–T6 (n = 8), T7–T8 (n = 8), T9–T10 (n = 8), T11–T12 (n = 8), (+ ribs)	T1–T2, T3–T4, T5–T6, T7–T8, T9–T10, T11–T12	5 Nm
Wilke et al. (2020)	2020	58 ± 5 (50–65)	8 f, 1 m	T2–T3 (n = 6), T6–T7 (n = 6), T10–T11 (n = 6)	T2–T3, T6–T7, T10–T11	2.5 Nm
Orbach et al. (2023)	2023	70 ± 11 (59–87)	5 f, 2 m	T1–L5 (n = 5), T3–L5 (n = 1), T4–L5 (n = 1) (+ rib cage)	T1–T12, T12–L1, L1–L5	8 Nm
Liebsch et al. (2024)	2024	36 (26–45)	2 f, 4 m	C7–S1 (+ rib cage, n = 6)	T1–L1, L1–S1, T1–T5, T5–T9, T9–L1	5 Nm
Muriuki et al. (2024)	2024	47 ± 14 (19–62)	8 f, 8 m	C2–T1 (n = 16)	C2–C3, … , C7–T1	1.5 Nm
Taverne et al. (2024)	2024	n.a.	n.a.	C0–C2 (n = 9)	C0–C1, C1–C2	2 Nm
Vogt et al. (2024a)	2024	46 (19–60)	4 f, 3 m	C4–T1 (n = 7)	C4–C5, … , C7–T1	1.5 Nm
Vogt et al. (2024b)	2024	28 (19–47)	2 f, 4 m	C4–C5 (n = 6)	C4–C5	2.5 Nm

### 3.2 Coupled lateral bending during primary flexion/extension

**FIGURE 2:**
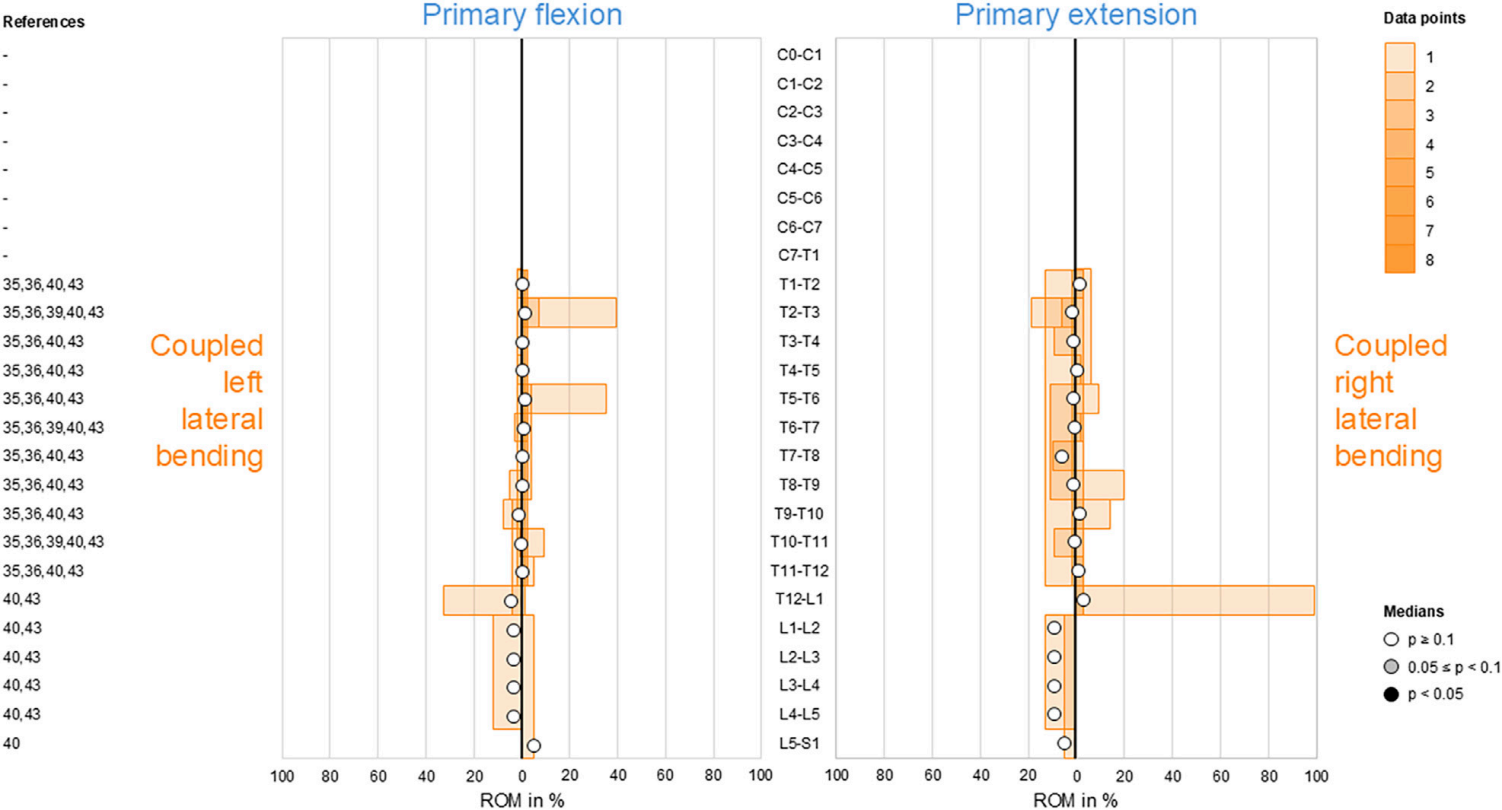
Bar diagrams illustrating segmental coupled lateral bending range of motion (ROM) relative to primary flexion/extension ROM acquired from *in vitro* studies (Liebsch et al., 2017; Liebsch et al., 2018; Wilke et al., 2020; Liebsch et al., 2024; Orbach et al., 2023).

There were no reported *in vitro* studies on coupled lateral bending during primary flexion/extension of the cervical spine (C0–T1). In the thoracic (T1–L1) and lumbar (L1–S1) spine, generally low and non-reproducible (*p* ≥ 0.1) lateral bending was found during primary flexion and primary extension (Figure 2).

### 3.3 Coupled axial rotation during primary flexion/extension

**FIGURE 3:**
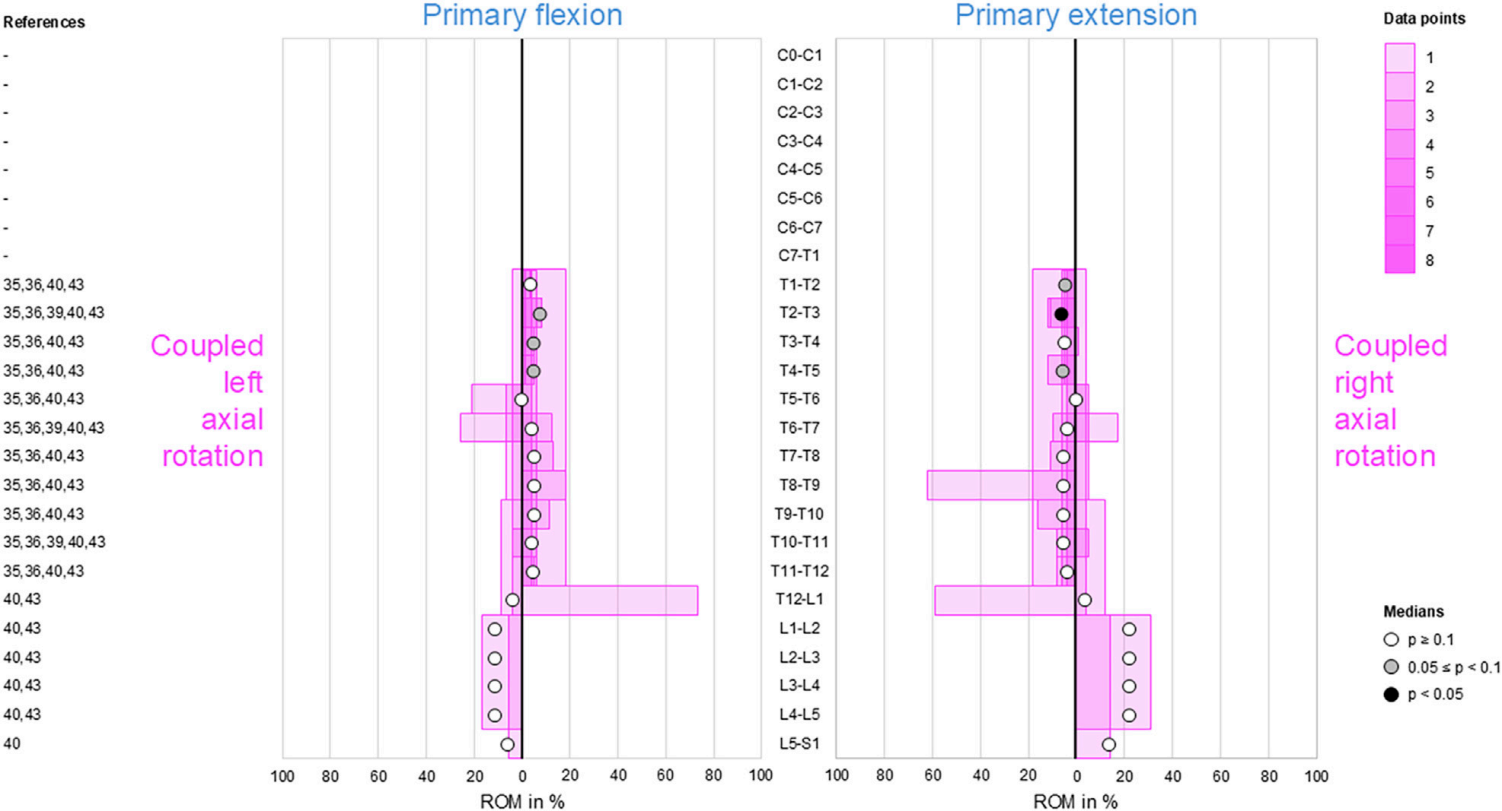
Bar diagrams illustrating segmental coupled axial rotation ROM relative to primary flexion/extension ROM acquired from *in vitro* studies (Liebsch et al., 2017; Liebsch et al., 2018; Wilke et al., 2020; Liebsch et al., 2024; Orbach et al., 2023).

For coupled lateral bending, there was no reported data on coupled axial rotation during primary flexion/extension for the cervical spine (C0–T1). Although coupled axial rotation was overall found to be low and non-reproducible (*p* ≥ 0.1) in the thoracic (T1–L1) and lumbar (L1–S1) spine, there were tendencies (0.05 ≤ *p* < 0.1) toward reproducible secondary right axial rotation during primary flexion (T2–T5) and secondary left axial rotation during primary extension (T1–T2, T4–T5) in the upper thoracic spine, with the latter even being reproducible (*p* < 0.05) at T2–T3 (Figure 3). Moreover, slightly moderate secondary right axial rotation during primary extension was noted in the lumbar spine (L1–L5), although it was non-reproducible (*p* ≥ 0.1).

### 3.4 Coupled flexion/extension during primary lateral bending

**FIGURE 4:**
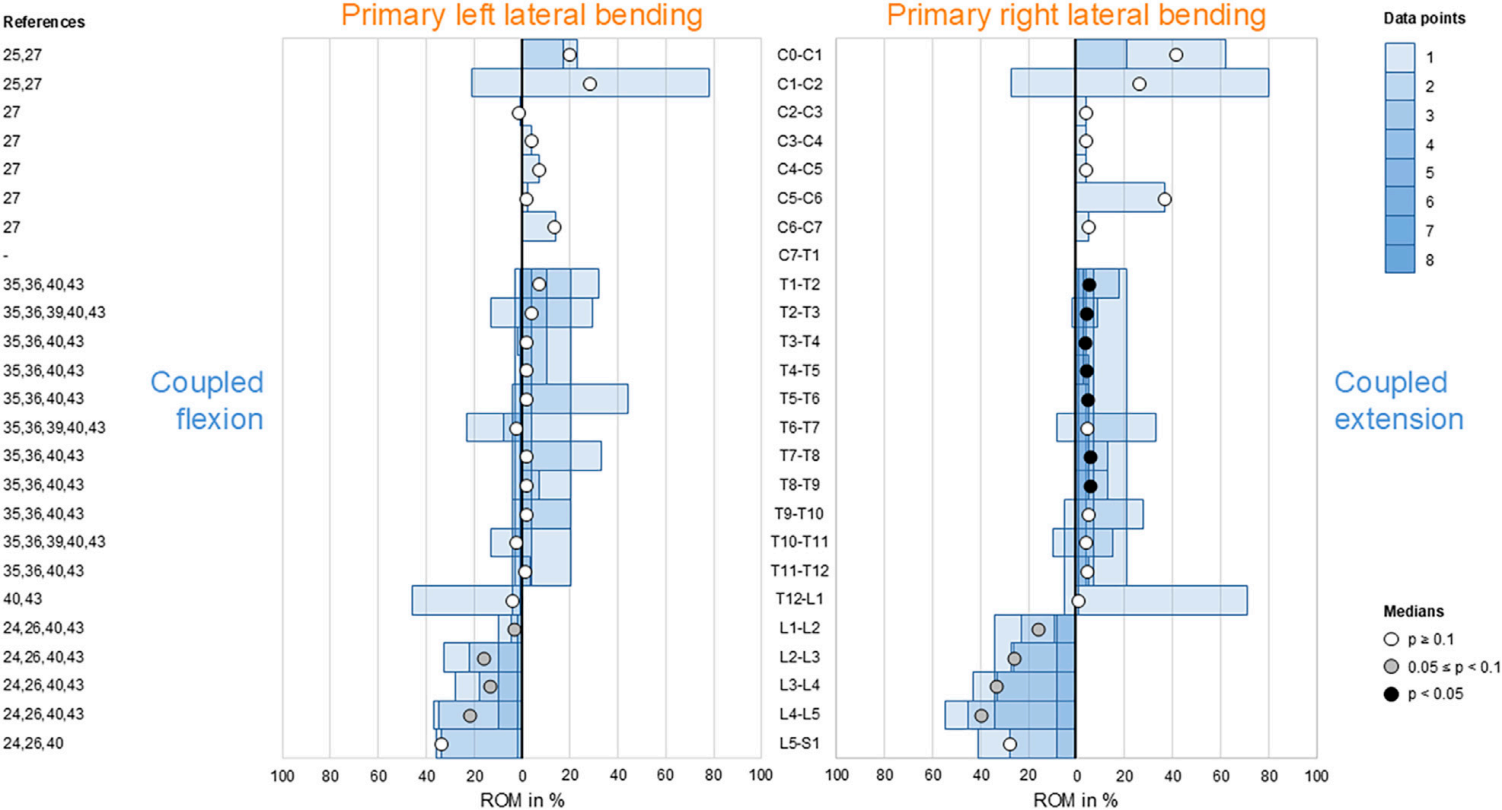
Bar diagrams illustrating segmental coupled flexion/extension ROM relative to primary lateral bending ROM acquired from *in vitro* studies (Panjabi et al., 1989; Panjabi et al., 1993a; Panjabi et al., 1994; Panjabi et al., 2001; Liebsch et al., 2017; Liebsch et al., 2018; Wilke et al., 2020; Liebsch et al., 2024; Orbach et al., 2023).

Secondary flexion and extension were found to be low in the thoracic spine (T1–L1) as well as low to moderate in the cervical (C0–T1) and lumbar (L1–S1) spine (Figure 4). However, there was a tendency (0.05 ≤ *p* < 0.1) toward reproducible coupled flexion during bilateral primary lateral bending in the lumbar spine (L1–L5). Moreover, reproducible (*p* < 0.05) coupled extension during primary right lateral bending was identified in the upper-thoracic (T1–T6) and mid-thoracic (T7–T9) spine.

### 3.5 Coupled axial rotation during primary lateral bending

**FIGURE 5:**
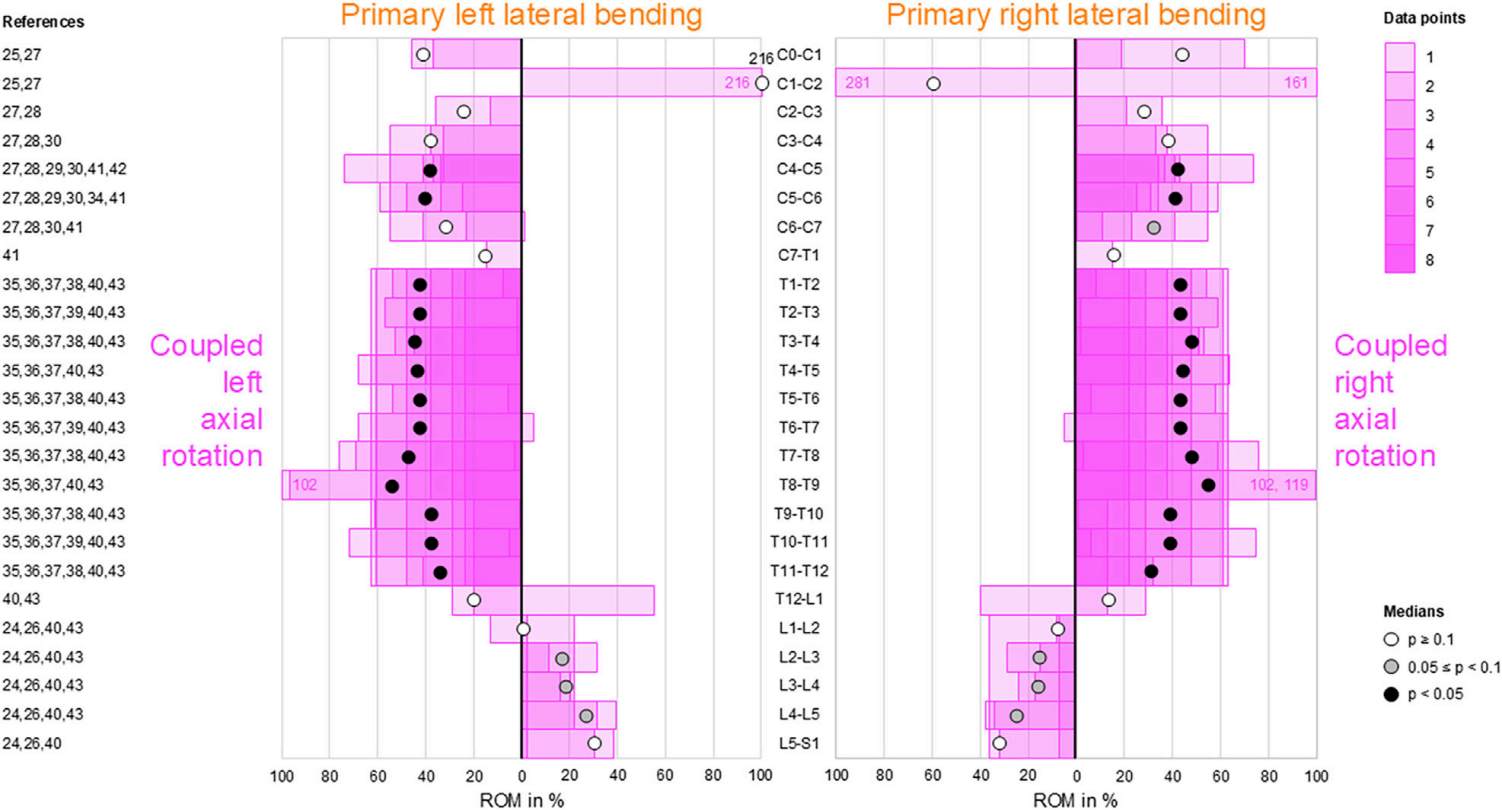
Bar diagrams illustrating segmental coupled axial rotation ROM relative to primary lateral bending ROM acquired from *in vitro* studies (Panjabi et al., 1989; Panjabi et al., 1993a; Panjabi et al., 1994; Panjabi et al., 2001; Wen et al., 1993; Barrey et al., 2009; Barrey et al., 2015; Colle et al., 2013; Liebsch et al., 2017; Liebsch et al., 2018; Liebsch et al., 2020b; Liebsch et al., 2020c; Wilke et al., 2020; Liebsch et al., 2024; Vogt et al., 2024a; Vogt et al., 2024b; Orbach et al., 2023).

Moderate ipsilateral axial rotations were found during primary lateral bending at the atlanto-occipital joint (C0–C1), in the subaxial cervical spine (C2–C7), and in the thoracic spine (T1–T12), indicating that primary left lateral bending was associated with moderate left axial rotation and primary right lateral bending was linked with moderate right axial rotation in these spinal sections (Figure 5). This coupled motion pattern was reproducible (*p* < 0.05) in the mid-cervical (C4–C6) and thoracic (T1–T12) spine. At the atlanto-axial joint (C1–C2), high to dominant contralateral axial rotation was detected during primary lateral bending but was non-reproducible (*p* > 0.1) as there were only two available data points, meaning that primary left lateral bending resulted in high-to-dominant right axial rotation and primary right lateral bending resulted in high-to-dominant left axial rotation in these spinal sections. The same pattern was found in the lumbar spine (L1–S1) with low (L1–L4) to moderate (L4–S1) coupled motions and a tendency (0.05 ≤ *p* < 0.1) toward reproducibility for the mid-lumbar segment motions (L2–L5).

### 3.6 Coupled flexion/extension during primary axial rotation

**FIGURE 6:**
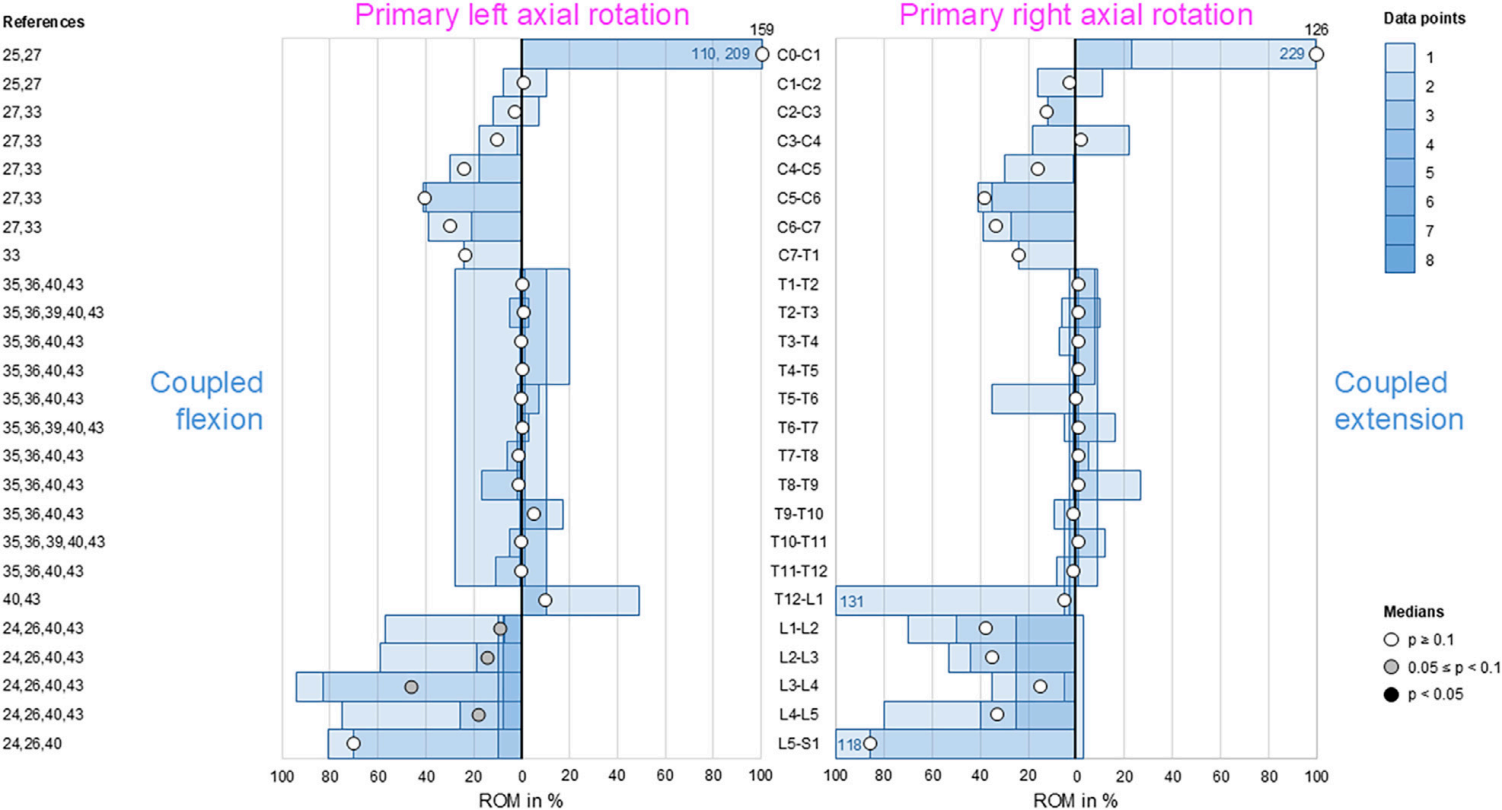
Bar diagrams illustrating segmental coupled flexion/extension ROM relative to primary axial rotation ROM acquired from *in vitro* studies (Panjabi et al., 1989; Panjabi et al., 1993a; Panjabi et al., 1994; Panjabi et al., 2001; Muriuki et al., 2024; Liebsch et al., 2017; Liebsch et al., 2018; Wilke et al., 2020; Liebsch et al., 2024; Orbach et al., 2023).

During primary axial rotation, secondary flexion and extension were low in the thoracic spine (T1–L1) and low to moderate in the cervical (C1–C2) and lumbar (L1–L5) spine. For these spinal sections, coupled flexion/extension was overall non-reproducible (*p* > 0.1) except for the lumbar spine (L1–L5), where there was a tendency (0.05 ≤ *p* < 0.1) toward secondary flexion during primary left axial rotation (Figure 6). High coupled flexion at the lumbosacral joint (L5–S1) and dominant coupled extension at the atlanto-occipital joint (C0–C1) were detected during bilateral primary axial rotation, both of which were non-reproducible (*p* > 0.1) owing to the low numbers of data points.

### 3.7 Coupled lateral bending during primary axial rotation

**FIGURE 7:**
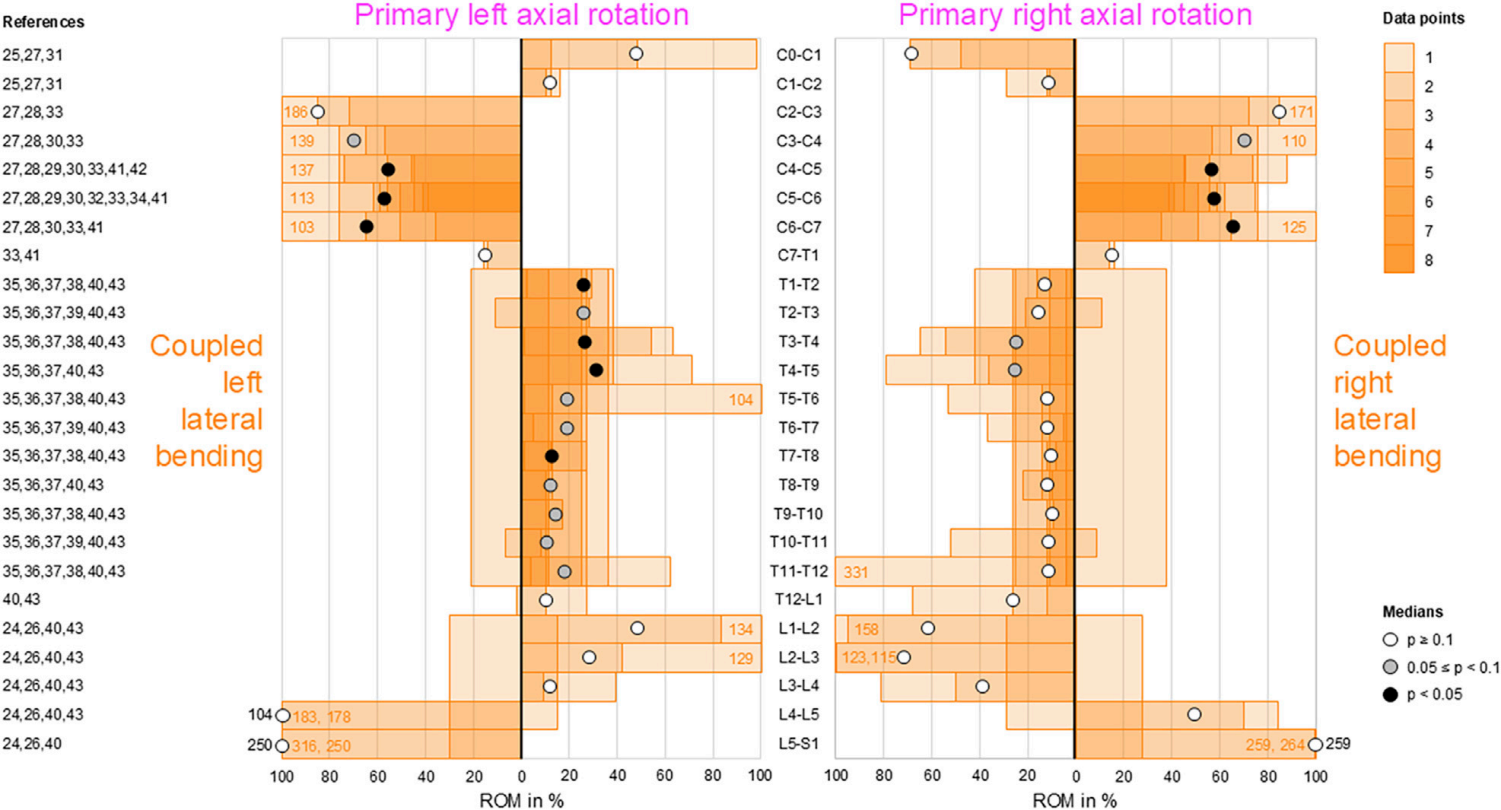
Bar diagrams illustrating segmental coupled lateral bending ROM relative to primary axial rotation ROM acquired from *in vitro* studies (Panjabi et al., 1989; Panjabi et al., 1993a; Panjabi et al., 1994; Panjabi et al., 2001; Wen et al., 1993; Barrey et al., 2009; Barrey et al., 2015; Taverne et al., 2024; Patwardhan et al., 2012; Muriuki et al., 2024; Colle et al., 2013; Liebsch et al., 2017; Liebsch et al., 2018; Liebsch et al., 2020b; Liebsch et al., 2020c; Wilke et al., 2020; Liebsch et al., 2024; Vogt et al., 2024a; Vogt et al., 2024b; Orbach et al., 2023).

Low-to-moderate contralateral lateral bending during primary axial rotation was detected in the upper (C0–C2) and lower (C7–T1) cervical, thoracic (T1–L1), and upper lumbar (L1–L4) spine, whereas high contralateral bending was found at the atlanto-occipital joint (C0–C1) and upper lumbar (L1–L3) segments during primary right axial rotation (Figure 7); these indicate that primary left axial rotation was associated with low-to-moderate right lateral bending and primary right axial rotation was linked with low-to-high left lateral bending in these spinal sections. However, reproducible (*p* < 0.05) contralateral lateral bending and tendency toward reproducible (0.05 ≤ *p* < 0.1) contralateral lateral bending were primarily determined for primary left axial rotations in the thoracic spine (T1–T12). High ipsilateral lateral bending during primary axial rotation was identified in the subaxial cervical spine (C2–C7) and dominant ipsilateral lateral bending was noted during primary axial rotation in the lower lumbar spine (L4–S1), meaning that primary left axial rotation was associated with high-to-dominant left lateral bending and primary right axial rotation was linked with high-to-dominant right lateral bending in these spinal sections. However, reproducible (*p* < 0.05) ipsilateral lateral bending during primary axial rotation was detected only in the lower cervical spine (C4–C7), and a tendency toward reproducible (0.05 ≤ *p* < 0.1) contralateral bending during primary axial rotation was noted only in the mid-cervical spinal segments (C3–C4).

## 4 Discussion

Coupled motions represent important biomechanical parameters as they can be used to interpret the three-dimensional *in vivo* kinematics as well as validate the experimental and numerical models of the spine. Moreover, knowledge on coupled motions could support the diagnosis of and assessment of treatment success in pathologies related to three-dimensional spinal mobility, such as degenerative disc disease, ankylosing spondylitis, and spinal deformities. In the specific case of scoliosis, better understanding of the coupled motions would be useful for guiding corrective movements through the use of braces and physical therapy. Although *in vivo* studies are limited in their extent of reproducibility owing to individual movement comforts of the participants and measurement imprecisions, *in vitro* studies under clearly defined and standardized loading conditions could provide more reproducible measurements to determine the segment- and section-specific coupled motions, even if only regarding the passive structures of the spine. Therefore, this meta-analysis of a large set of *in vitro* data from literature was able to identify characteristic coupled motion patterns of the entire spine in the main anatomical motion directions.

**FIGURE 8:**
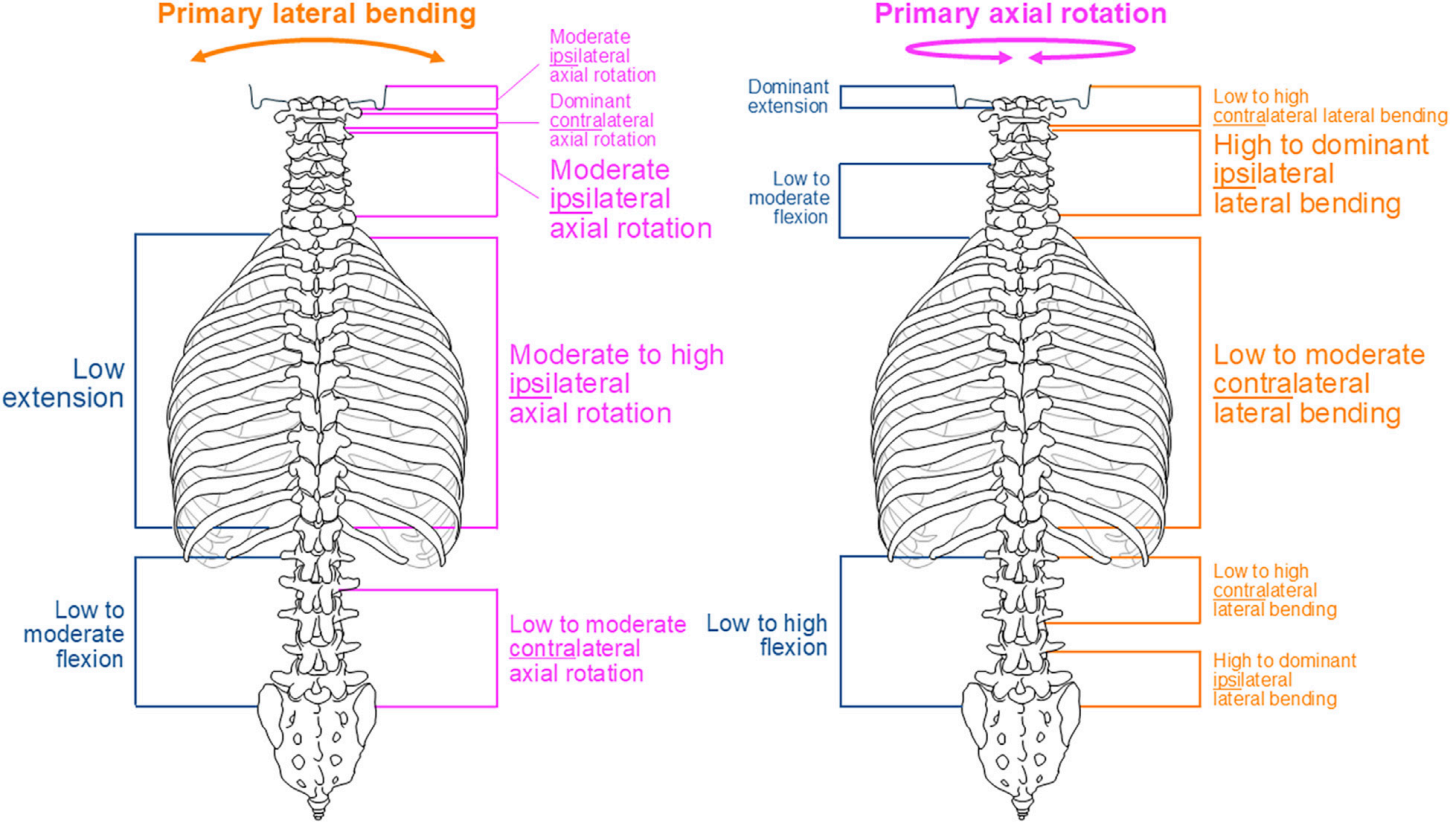
Overview of the main findings of this meta-analysis regarding coupled motion patterns during primary lateral bending (left) and primary axial rotation (right). Consistent but non-significant findings are depicted in small font, findings tending overall toward reproducibility are shown in medium font, and overall reproducible findings are depicted in large font.

Although complex and section-specific relationships were found between the different primary and secondary motions in this meta-analysis, the overall symmetrical coupled motion characteristics with regard to the sagittal plane were determined for individual primary motion planes. These findings may also explain the low and non-reproducible coupled motions noted during primary flexion/extension as the normal non-deformed spine is almost symmetrical with regard to the sagittal plane. However, this meta-analysis also revealed direction-specific differences in the reproducibility of coupled motions, particularly for the more reproducible coupled extension during primary right lateral bending (Figure 4) and more reproducible contralateral right lateral bending during primary left axial rotation (Figure 7) in the thoracic spine. As these observations are contrary to the expected symmetric coupled motions owing to the (ideal) anatomical symmetry of the spine, future studies should additionally evaluate the effects of three-dimensional spinal curvature when determining spinal coupled motions. While the *in vitro* studies evaluated in this systematic review explicitly excluded spinal specimens exhibiting major deformities, the findings may nevertheless be relevant for the clinical interpretation of early scoliosis. Although some of the results do not completely correspond with common clinical findings, such as the more reproducible coupled extension during primary right lateral bending in contrast to the frequently observed trend toward a flat back for both left and right thoracic curvatures, many of the findings show high correspondence, such as the low coupled sagittal plane motions and ipsilateral axial rotation in the thoracic spine or progressive loss of lordosis in the lumbar spine during primary lateral bending, as well as the characteristic associations between contralateral bending, apical derotation of thoracic scoliosis toward the concavity, and reversed biomechanical effects at the lumbar level. Thus, our meta-analysis confirmed the strong motion coupling relationships between lateral bending and axial rotation in the healthy spine that were previously reported in early investigations by Lovett (1900); Lovett, (1905) and have been confirmed in numerous subsequent *in vivo*, *in vitro*, and *in silico* studies. However, the previous works often report conflicting findings regarding the extent and quality of coupled motions for individual spinal sections or segment levels, whereas our meta-analysis summarizes specific coupled motion patterns of the entire spine in a quantitative (Figures 2–7) as well as qualitative (Figure 8) manner based on a large dataset.

This meta-analysis reveals shifts in the coupled motion directions at specific anatomical points, especially the thoracolumbar transition (T12–L2) during primary lateral bending (Figures 4, 5) and cervicothoracic transition (C7–T2) during primary axial rotation (Figures 6, 7); furthermore, transitions are observed in the atlanto-occipital joint (C0–C1), atlanto-axial joint (C1–C2), and upper subaxial spine (C2–C3) during primary lateral bending and primary axial rotation (Figures 4–7). These shifts in the directions of the coupled motions may be explained by the specific changes in the anatomical features among these spinal areas, particularly the morphology and orientation of the facets as well as spinal curvature within the sagittal plane in the case of the subaxial cervical, thoracic, and lumbar spine. Using a computational model of the thoracic and lumbar spine, Schultz et al. (1973) showed in an early work that spinal motion coupling is a function of segment orientation; later *in silico* investigations reported by Scholten and Veldhuizen (1985) confirmed the impact of sagittal plane curvature on the coupled motion behaviors of the spine, further revealing the effects of the facet angles in the transverse and sagittal planes. Indeed, an anatomical study of the three-dimensional orientations of the facets by Panjabi et al. (1993b) showed that the facet width, height, and width/height ratio as well as the transverse and sagittal plane angles of the facets change abruptly at the cervicothoracic and thoracolumbar transitions, matching the findings of this meta-analysis well. Previous *in vitro* studies on the thoracic spine did not report any significant coupled motions in monosegmental and bisegmental specimens (Liebsch et al., 2020c; Wilke et al., 2020; Greiner-Perth et al., 2025), substantiating the effect of the sagittal curvature on the emergence of coupled motions. Furthermore, other anatomical structures like the ligaments (Wilke et al., 2020) and rib cage (Brasiliense et al., 2011; Liebsch et al., 2017; Liebsch et al., 2020c; Liebsch and Wilke, 2022) were shown to not cause alterations in the coupled motion behaviors of the thoracic spine during resection or like the intervertebral disc during isolation (Wilke et al., 2020).

Coupled motions of the spine are often equated with and used synonymously for coupled rotations of the spine in literature because coupled translations of the spine are often neglected when considering secondary motions. Indeed, coupled translations were only investigated in two of the 20 *in vitro* studies evaluated herein (Panjabi et al., 1994; Panjabi et al., 2001). For the cervical spine, Panjabi et al. (2001) applied pure moments of 1 Nm and found a highly coupled anterior translation of about 10 mm during primary flexion and a highly coupled posterior translation of about 10 mm during primary extension at the atlanto-axial joint (C1–C2), a moderate ipsilateral translation of up to 4 mm during bilateral lateral bending at the atlanto-axial joint (C1–C2) and upper subaxial spine (C2–C3), and a highly coupled posterior translation of up to 12 mm at the atlanto-occipital (C0–C1) and atlanto-axial (C1–C2) joints during bilateral axial rotation. To determine the single motion segments of the lumbar spine (L1–S1), Panjabi et al. (1994) applied pure moments of 10 Nm and detected moderately coupled superior translations of up to 4 mm and low-coupled anterior translations of up to 2 mm during primary flexion, along with low-coupled inferior and posterior translations of up to 2 mm during primary extension and low ipsilateral translations of up to 1 mm during left and right lateral bending. Although extensive investigations were performed in these two studies, the available data on coupled translations is still limited, necessitating additional studies in the future.

Despite our attempt to present a comprehensive meta-analysis, this work has several limitations. For the data analyses, coupled motion data of single segmental levels were combined with coupled motion data spanning multiple segmental levels, potentially resulting in underestimation of the coupled motions compared to evaluation of only polysegmental data owing to the enhancing effect of the increasing sagittal plane curvature. This approach was inevitable for increasing the sample size per segmental level and minimizing the effects of outliers to create as statistically significant results as possible. In fact, even with our approach, there were few or even no available data points for some segmental levels and primary motion directions, particularly for the entire cervical spine with regard to primary flexion/extension. As larger sample sizes per segmental level may be assumed to entail higher statistical power of the findings, more *in vitro* data from different investigations are required to substantiate the findings of this meta-analysis, especially for the cervical and lumbar spinal regions for which moderate-to-dominant but non-reproducible coupled motions were found. Moreover, combining coupled motion data from studies with different numbers of tested segmental levels may result in distortions of the extent and quality of the coupled motions, such as those found for coupled axial rotation during primary lateral bending at the atlanto-axial joint (C1–C2) (Figure 5); these observations were based on one investigation with C0–C3 specimens (Panjabi et al., 1993a) and another investigation with C0–C7 specimens (Panjabi et al., 2001) reported by the same research group using the same loading conditions. Similarly, one study reported the opposite coupled lateral bending during primary axial rotation for the thoracic spine (T1–T12) compared to most of the prior studies (Figure 7) when using the entire thoracic to lumbar spine segments (T1–L5) (Orbach et al., 2023). Although the application of pure moments should theoretically create the same primary and coupled motions in monosegmental and polysegmental specimens, the overall coupled motion behaviors may be affected by the number of contiguous segmental levels when different segmental coupled motion characteristics are tested together. Moreover, we only included *in vitro* studies that used pure moments in this meta-analysis to ensure the highest possible data comparability. Owing to this approach, *in vitro* studies that used additional axial compressive or follower loads to simulate bodyweights and muscle forces were not included, even though it was shown that follower loading can increase the extent of coupled motions (Liebsch et al., 2018). This phenomenon might be explained by the loss of intervertebral disc flexibility, which could potentially increase the effect of sagittal plane curvature when assuming the spine as a torsionally stiff and sagittally curved rod, and the enhanced facet surface contact, which would support the facets in acting as three-dimensionally inclined guide rails. Consequently, the non-consideration of axial compressive or follower loading might entail underestimation of the coupled motions compared to pure-moment loading. Moreover, active muscle control affects the *in vivo* coupled motions that cannot be replicated by *in vitro* studies using pure moments, as summarized in this meta-analysis on the coupled motions of passive spinal structures. Another limitation of this meta-analysis is that the primary and secondary ROMs of single motion segments are often low, which could potentially cause large variations in the relative coupled motion data. Lastly, the standard deviations and ranges of the data in the original studies were not included in this meta-analysis as these values were often not reported or exhibited high variations. Nevertheless, the strength of this meta-analysis in aggregating quantitative data acquired from *in vitro* studies using standardized loading conditions might outweigh these limitations with regard to the high comparability of spinal coupled motion data.

## 5 Conclusion

This meta-analysis of *in vitro* studies identifies characteristic coupled motion patterns of the spine. Although we did not determine considerable coupled motions for primary flexion/extension, there was evidence for motion coupling relationships between lateral bending and axial rotation. Specifically, primary lateral bending *inter alia* resulted in moderate-to-high ipsilateral axial rotation of the subaxial cervical and thoracic spine, whereas primary axial rotation *inter alia* caused low-to-moderate contralateral lateral bending at the thoracic spine and high-to-dominant ipsilateral lateral bending at the subaxial cervical spine. Despite the limited availability of data points that would require additional studies to extend and substantiate the findings of this meta-analysis, the collection of inhomogeneous data containing both monosegmental and polysegmental data as well as the inclusion of only studies using standardized but non-physiological loading conditions in our dataset will be valuable for validating both experimental and numerical studies of the spine as well as interpreting the coupled motion behaviors of passive spinal structures.

## Data Availability

The original contributions presented in the study are included in the article/supplementary material, and any further inquiries may be directed to the corresponding author.
